# Male-biased recombination at chromosome ends in a songbird revealed by precisely mapping crossover positions

**DOI:** 10.1093/g3journal/jkae150

**Published:** 2024-07-10

**Authors:** Hongkai Zhang, Max Lundberg, Suvi Ponnikas, Dennis Hasselquist, Bengt Hansson

**Affiliations:** Department of Biology, Lund University, 22362 Lund, Sweden; Department of Biology, Lund University, 22362 Lund, Sweden; Department of Biology, University of Oulu, 90570 Oulu, Finland; Department of Biology, Lund University, 22362 Lund, Sweden; Department of Biology, Lund University, 22362 Lund, Sweden

**Keywords:** recombination, crossover, male bias, sub-telomeric regions, SNP, songbird, great reed warbler

## Abstract

Recombination plays a crucial role in evolution by generating novel haplotypes and disrupting linkage between genes, thereby enhancing the efficiency of selection. Here, we analyze the genomes of 12 great reed warblers (*Acrocephalus arundinaceus*) in a 3-generation pedigree to identify precise crossover positions along the chromosomes. We located more than 200 crossovers and found that these were highly concentrated toward the telomeric ends of the chromosomes. Apart from this major pattern in the recombination landscape, we found significantly higher frequencies of crossovers in genic compared with intergenic regions, and in exons compared with introns. Moreover, while the number of recombination events was similar between the sexes, the crossovers were located significantly closer to the ends of paternal compared with maternal chromosomes. In conclusion, our study of the great reed warbler revealed substantial variation in crossover frequencies within chromosomes, with a distinct bias toward the sub-telomeric regions, particularly on the paternal side. These findings emphasize the importance of thoroughly screening the entire length of chromosomes to characterize the recombination landscape and uncover potential sex-biases in recombination.

## Introduction

Recombination has profound evolutionary implications by generating new haplotypes that natural selection can act upon. The process of reshuffling haplotypes through recombination breaks linkage disequilibrium (LD) and reduces the interference between linked loci, which otherwise limits the action of natural selection (Felsenstein 1974; Otto 2021). Recombination disconnects beneficial and deleterious alleles at linked loci, facilitating adaptive evolution (increasing the frequency of advantageous alleles) and purging the genetic load (reducing the frequency of deleterious alleles). The Y and W chromosomes of mammals and birds illustrate the importance of recombination, as their prolonged periods without recombination have resulted in significant degeneration and paucity of genes (Charlesworth and Charlesworth 2000; Bachtrog 2013).

Recombination not only has evolutionary implications but can also be subject to selection and undergo evolutionary changes itself. Studies have demonstrated variation in recombination rates across clades, species, populations, and between sexes (Ritz *et al*. 2017; Stapley *et al*. 2017; Peñalba and Wolf 2020). For example, fungi generally exhibit higher rates of recombination (averaging 48.7 cM/Mb) compared with plants (averaging 1.9 cM/Mb) (Stapley *et al*. 2017), and among mammals, recombination rates range from 0.2 cM/Mb in opossums to 1.6 cM/Mb in dogs (Dumont and Payseur 2008). The variation in recombination rate across species and deeper lineages can be attributed, at least in part, to evolved differences in chromosome size and number. The presence of more and smaller chromosomes tends to increase recombination rates since each chromosome (or chromosome arm) requires a minimum of 1 crossover (CO) on one of the 2 sister chromatids (Coop and Przeworski 2007; Stapley *et al*. 2017). Regarding sex differences, the most extreme scenario is when recombination is entirely absent in 1 sex (achiasmy). This lack of recombination coincides almost exclusively with the heterogametic sex, such as male *Drosophila* (XY) and female Lepidoptera (ZW) (Haldane 1922; Huxley 1928; John *et al*. 2016). However, in many plants and animals, the degree of sexual dimorphism in recombination (heterochiasmy) is less pronounced, with at least some level of recombination in both sexes (Burt *et al*. 1991; Broman *et al*. 1998; Maddox *et al*. 2001; Berset-Brändli *et al*. 2008; Wellenreuther *et al*. 2013; Bergero *et al*. 2019; Malinovskaya *et al*. 2020). Generally, it is not surprising that recombination can evolve since it exhibits typical features of evolving traits, such as variation among individuals and a heritable component, as seen in humans (Kong *et al*. 2004) and sheep (Johnston *et al*. 2016). In fact, evidence from experimental populations in *Drosophila* shows that the rate of recombination can be manipulated over short evolutionary time scales (Aggarwal *et al*. 2015; Kohl and Singh 2018).

Quantifying the recombination landscape—the local recombination rate variation along the chromosomes—can provide valuable insights into the influence of recombination on various biological processes. For instance, as selection operates on chromosome regions (linked selection), low recombining regions often exhibit reduced selection efficiency at single mutations, lower local effective population size (N_e_), and stronger genetic drift (Peñalba and Wolf 2020). Moreover, recombination can contribute to elevated GC content through GC-biased gene conversion (Mugal *et al*. 2015) and increase genetic variation by locally raising the mutation rate (Filatov and Gerrard 2003; Hellmann *et al*. 2003; Huang *et al*. 2005; Arbeithuber *et al*. 2015; Halldorsson *et al*. 2019). As expected, the local recombination rate correlates with various population genetic parameters, including LD, nucleotide diversity, GC and repeat content, and gene density (Peñalba and Wolf 2020; Ponnikas *et al*. 2022). A common pattern observed in many species is an increase in recombination rates toward the telomeres and decreasing rates around the centromeres (Vincenten *et al*. 2015; Limborg *et al*. 2016; Haenel *et al*. 2018; Sardell and Kirkpatrick 2020). Mechanistically, heterochromatin, which is enriched around the centromeres, can prevent local recombination by hindering polymerase accessibility or through repression of double-strand break (DSB) formation caused by methylation, RNAi or specific enzymes (Ellermeier *et al*. 2010; Melamed-Bessudo and Levy 2012; Wijnker *et al*. 2013). Quantifying the recombination landscape allows the identification of recombination hotspots where recombination is particularly frequent, as well as cold spots, where recombination is infrequent. Notably, detailed analysis of recombination hotspots in humans and mice led to the discovery of the *PRDM9* gene as a key regulator of the local recombination rate (Myers *et al*. 2008; Baudat *et al*. 2010). However, while *PRDM9* is present in most mammals, it is absent in birds, suggesting different mechanisms of recombination regulation across taxa (Singhal *et al*. 2015). Furthermore, there are still many unanswered questions, such as why and how the recombination landscapes sometimes differ between sexes, given the observation of higher recombination rates closer to the chromosome ends in males and more uniform recombination landscapes in females (Sardell and Kirkpatrick 2020). Exploring these differences and understanding their underlying mechanisms remain areas of active research.

The initial data of recombination came from direct observations of chiasmata and from segregation of Mendelian traits in model organisms, such as *Drosophila* (Morgan 1910; Haldane 1920; Morton *et al*. 1976). Subsequently, segregation analysis of molecular markers in large pedigrees became popular for constructing linkage maps and inferring recombination distances in centimorgans (cM) (Robinson 1996; Dumont *et al*. 2011). Combining physical maps and linkage maps allowed infering local recombination rates along chromosomes (Groenen *et al*. 2009; Backström *et al*. 2010; Kawakami *et al*. 2014; Johnston *et al*. 2017). The advent of next-generation sequencing (NGS) enabled the screening of dense marker sets in many individuals, facilitating pedigree-free methods to study recombination rates. These methods involve assessing the local level of LD and assuming a negative correlation between LD and recombination rate (Singhal *et al*. 2015; Provost *et al*. 2022). This has been accompanied by progress in statistics and model inferences of the population-level recombination landscape (McVean and Auton 2007; Chan *et al*. 2012; Gao *et al*. 2016; Adrion *et al*. 2020). Furthermore, dense marker data provided by NGS allows locating individual CO positions with high precision on chromosomes by phasing alleles from their segregation pattern in small pedigrees (Smeds *et al*. 2016), or, as applied most recently, by directly generating haploid data through sperm sequencing (Bell *et al*. 2020). In birds, high-resolution recombination positioning using NGS-generated single nucleotide polymorphism (SNP) data has so far been applied to a collared flycatcher (*Ficedula albicollis*) 3-generation pedigree (Smeds *et al*. 2016).

In this study, we aim to identify recombination positions at a highly detailed chromosomal scale by analyzing genome-wide SNPs segregating in a 3-generation pedigree of the great reed warbler (*Acrocephalus arundinaceus*). To achieve this, we sequenced the genomes of the individuals in the pedigree, mapped the reads to the reference genome of the great reed warbler (Sigeman *et al*. 2021), and called SNPs. Then, we employed the newly developed *RecView* R package (Zhang and Hansson 2023) to analyze the genome-wide SNP data, allowing us to examine the segregation patterns at each SNP in the pedigree and pinpoint recombination positions between genomic regions inherited from different grandparents. Previous studies have indicated significant heterochiasmy in the great reed warbler, with females exhibiting nearly twice the recombination rate compared with males (Hansson *et al*. 2005; Dawson *et al*. 2007). However, these earlier studies were limited by the use of few markers covering only small portions of the chromosomes. In contrast, our present study utilizes millions of SNPs, which enables us to examine whether recombination events preferentially occur in specific regions of the chromosomes and whether the number and positions of recombination events differ between paternal and maternal chromosomes.

## Materials and methods

### Generating the SNP dataset

**Fig. 1.**
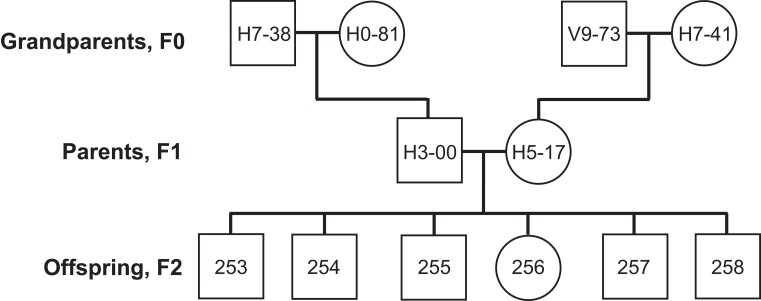
The 3-generation great reed warbler pedigree analyzed in the present study. Project-specific individual codes are given. Squares represent males and circles represent females.

The great reed warbler is a large Acrocephalid warbler and long-distance migrant that spends the winter in sub-Saharan Africa, and returns to breed in reed lakes in Europe and Western Asia during the summer (Helbig and Seibold 1999; Lemke *et al*. 2013; Koleček *et al*. 2016; Sjöberg *et al*. 2021). We selected a 3-generation pedigree from our long-term study population of great reed warblers at Lake Kvismaren, Sweden (Bensch *et al*. 1998; Hasselquist 1998; Tarka *et al*. 2014; Hansson *et al*. 2018). The pedigree consisted of 12 individuals (Fig. 1) and included 4 grandparents (F0 generation), 2 parents (F1 generation) and 6 offsprings (F2 generation), with the offspring belonging to the 1998 cohort (5 males and 1 female; Supplementary Table 1).

We used a phenol–chloroform protocol to extract genomic DNA from blood stored in SET buffer (0.015 M NaCl, 0.05 M Tris, 0.001 M EDTA, pH 8.0) of each of the 12 individuals. Sequencing libraries were created using the TruSeq (Illumina) protocol with 350 bp insert size, and sequenced on a NovaSeq 6000 (Illumina) using a 2 × 150 bp setup and targeting 50 × coverage. Libraries and sequencing were performed by SciLifeLab, Uppsala, Sweden.

The raw sequence reads were trimmed with *trimmomatic* version 0.39 (Bolger *et al*. 2014), mapped to the reference genome assembly of the great reed warbler (Sigeman *et al*. 2021) using *bwa mem* version 0.7.17 (Li 2013), and read duplicates were removed with *PicardTools* version 2.27.5 (Broad Institute). Then, variants were called with *freebayes* version 1.3.2 (Garrison and Marth 2012), producing a VCF file. Variants in annotated repeat intervals were removed using *vcftools* version 0.1.16 (Danecek *et al*. 2011). Only bi-allelic variants were kept, and decomposed for complex and multinucleotide variants, using *vcftools* and *VT decompose_blocksub* version 0.5 (Tan *et al*. 2015). After indels had been removed using *vcffilter* from *vcflib* version 2017-04-04 (Garrison *et al*. 2022), the SNPs were divided into an autosome and a Z-linked dataset. Filtering on quality, strandedness, read placement and genotype coverage (with -f “QUAL > 30 & SAF > 0 & SAR > 0 & RPR > 0 & RPL > 0” and -g “DP > 9” in *vcffilter*) were applied for both datasets. Finally, SNPs with missing data were removed using *vcftools*, leading to 6,650,271 autosomal SNPs, 9,177 SNPs on PAR and 195,250 Z-linked SNPs.

### Chromosome-level assembly and chromosome arms

The genome assembly of the great reed warbler consists of relatively few large scaffolds (Sigeman *et al*. 2021). Some of the smaller chromosomes are represented by a single scaffold (chromosomes 4A, 13, 14, 19, 20, 23, and 30; Sigeman *et al*. 2021), and the Z chromosome (which includes a translocated part of chromosome 4A) has previously been assembled to chromosome-level (Ponnikas *et al*. 2022). The remaining chromosomes are represented by between 2 and 9 scaffolds (Sigeman *et al*. 2021). To ordered and oriented the scaffolds of the remaining chromosomes, we used synteny analyses to several sources. We used the chromosome-level assemblies of the zebra finch (*Taeniopygia guttata*; bTaeGut1.4.pri, NCBI BioProject ID PRJNA489098) and the great tit (*Parus major*; Parus_major1.1, NCBI BioProject ID PRJNA208335) to assign scaffolds to chromosomes. Next, the scaffolds were ordered and oriented using de novo assemblies of 2 additional great reed warbler individuals (1 male and 1 female; neither being the individual used for the original assembly; constructed with 10X linked read and HiC data) (B. Hansson *et al.*, unpubl. data) and the chromosome-level assembly of another *Acrocephalus* species, the Eurasian reed warbler (*A. scirpaceus*; Sætre *et al*. 2021). During this process, we detected that the scaffold Contig1 on chromosome 2 was wrongly assembled, and this was corrected (Supplementary Table 2). When the 2 de novo assemblies gave contradictory suggestions about the order or orientation (which could happen for methodological and biological reasons, such as the presence of inversion polymorphisms), we selected the order or orientation that was supported by the Eurasian reed warbler assembly and/or the recombination analysis in this study (Supplementary Table 2). We did not manage to include some of the micro-chromosomes and chromosome 16 (the latter contains the structurally complicated major histcompatibility complex; Westerdahl *et al*. 2022). Moreover, 1 chromosome was deemed too short for reliable analysis of recombination (chromosome 30; 1.3 Mb) and 1 had several scaffolds with uncertain order and orientation (chromosome 22; see below). Thus, the final number of chromosomes assembled to chromosome-level and included in the present analysis was 29 autosomes and the Z chromosome (Supplementary Table 2), which is similar to that of the great tit and Eurasian reed warbler genome assemblies, but lower compared with the zebra finch where all autosomes are (at least partly) assembled (bTaeGut1.4.pri; NCBI BioProject ID PRJNA489098). We named the chromosomes according to their zebra finch homologs (this study and Sigeman *et al*. 2021). The total length of the autosomal assembly was 978 Mb, which is similar to the great tit and zebra finch assemblies (1,020 and 1,056 Mb, respectively).

Autosomes 1, 1A, 2, 3, and 4 are sub-telocentric or sub-metacentric (i.e. have 2 chromosome arms) in the zebra finch, whereas the remaining autosomes are telocentric (Knief and Forstmeier 2016). For these sub-telocentric or sub-metacentric chromosomes, we estimated the approximate location of the centromere based on low nucleotide diversity among the 12 sequenced individuals (extremely low nucleotide diversity is expected in the centromeric regions; Stump *et al*. 2005), and its location in the zebra finch (Knief and Forstmeier 2016), and divided them into 2 arms with the longer arm denoted q and the shorter p (Supplementary Table 3). The Z chromosome is acrocentric in the zebra finch (Knief and Forstmeier 2016), but we did not divide this chromosome in the great reed warbler as there is an exceptionally low nucleotide diversity in a large central part of the great reed warbler Z chromosome (the region between c. 15–70 Mb; total length 87.5 Mb; Ponnikas *et al*. 2022). However, the Z chromosome has a small pseudoautosomal region (PAR), a region that recombines with the W chromosome in females. As we have 1 female offspring in our dataset, which has inherited a W chromosome from its mother, we present data for the PAR (1.1 Mb; where both sexes may recombine) and the non-PAR (86.4 Mb; where only males recombine) of the Z chromosome, separately.

### Localizing crossovers using the *RecView* R package

For viewing and locating crossovers (COs), we used *RecView*, an R package that we recently developed specifically for segregation analysis of SNPs in 3-generation pedigrees (Zhang and Hansson 2023). *RecView* analyses the grandparent-of-origin of alleles at each SNP in each offspring (F2 generation in the pedigree) and identifies COs in the boarders between chromosome regions inherited from the paternal and maternal grandparents, respectively. *RecView* requires 2 input files, one providing the order and orientation of the scaffolds of the reference genome, and one providing the genotype data of the individuals. The genotype files for the autosomes and the Z chromosome were generated by extracting genotypes from the VCF files using *vcftools* (command: vcftools –gzvcf [vcf file] –extract-FORMAT-info GT –stdout > [output file]), and converting the data using the *make_012gt()* function in *RecView* (for a description of the input files, see Zhang and Hansson 2023).

In the *RecView*, we seleted the Cumulative Continuity Score (CCS) algorithm with a threshold of 50 (represented with CCS50) to locate the positions of putative COs (for a description of CCS, see Zhang and Hansson 2023). *RecView* also provides an estimate of the precision of the putative COs based on the local density of alleles with information of grandparent-of-origin. We conducted a manual examination of all CO positions and detected a few artifacts where identical CO positions occurred in more than 1 offspring and always coincided with scaffold borders. We strongly suspect that this indicates scaffolds that had been wrongly ordered or oriented, and therefore corrected and recalculated the CO positions accordingly (most of these were small and some of them were also supported by the Eurasian reed warbler assembly; corrected order and orientation are given in Supplementary Table 2). However, chromosome 22 had 2 such problematic scaffolds (contig83 and contig39_split_69700), and since we could not order and orient these scaffolds reliably, we excluded this chromosome from the analysis (Supplementary Table 2). Thus, our analyses of CO positions included 34 autosomal arms (29 autosomes, 5 with 2 arms) and the PAR and non-PAR regions of the Z chromosome (Supplementary Table 4).

We did not analyze gene conversions (i.e. non-CO recombination events) because they are expected to be very short (a few hundred base pairs; Duret and Galtier 2009; Gergelits *et al*. 2021), which means that a majority would likely be missed or represented by very few SNPs (as SNP density is insufficient). Additionally, as such events would be represented by very few SNPs, they are difficult to distinguish from genotype errors (e.g. caused by sequencing errors).

### Statistical tests

For each autosomal arm, we calculated both the sex-average and sex-specific recombination distance (cM) by dividing the number of paternal and maternal COs in the 6 offsprings with the number of analyzed meiosis (12 in total; 1 in each parent for each offspring) and multiplying the value with 100. The recombination distance in cM indicates the probability of observing a recombination event over a specific chromosome region (here, a chromosome arm). Next, the sex-average and sex-specific recombination distances were divided by the size of each autosomal arm to obtain respective recombination rates (cM/Mb). For the Z chromosome, the calculations of recombination distance and rate were the same as for the autosomes, but here we separated between the PAR where both parents may recombine and the rest of the chromosome where only the father may recombine.

We tested the difference between sexes in autosomal recombination distance (cM), and in autosomal recombination rate (cM/Mb), with paired t-tests on the autosomal arm-level using the *rstatix* R package (Kassambara 2022). Next, we tested whether CO positions differed between sexes by using both the actual and the proportional positions of COs on the chromosome arms (measured from the telomeric end of the chromosome arms) with a Mann–Whitney *U* test in the *stats* R package (R Core Team 2022).

We used available gene annotations (Sigeman *et al*. 2021) in *bedtools* (Quinlan and Hall 2010) to explore the overlap between autosomal COs and intergenic, genic, exon, intron, UTR, and CDS regions. We performed this analysis on 2 levels: (1) for the entire genome and (2) within the 6-Mb telomeric ends of the chromosomes (because most COs were located within these chromosomal regions (87%; see Results) and because these regions differ from other parts of the chromosomes in, for example, gene density). We tested whether the number of autosomal COs differed between the following pairs of annotation features: intergenic vs genic regions, exon vs intron, and UTR vs CDS. We used chi-squared goodness-of-fit tests to test whether the observed numbers of COs in each type of feature (e.g. exon and intron) differed from what would be expected from the total length (in bp) of the intervals of each feature, using the *rstatix* R package (Kassambara 2022). We used a permutation approach to test whether the locations of COs were closer to genes than expected by chance. To this end, we used *bedtools* to get a randomized position within each of the intergenic intervals where a CO was detected (N = 109) and calculated the median distance to the closest gene among all randomized intervals. This procedure was repeated 1,000 times to obtain a null distribution.

We used *tidyverse* R package (Wickham *et al*. 2019) for data handling, and plotted the results with *ggplot2* (Wickham 2011).

## Results

**Fig. 2.**
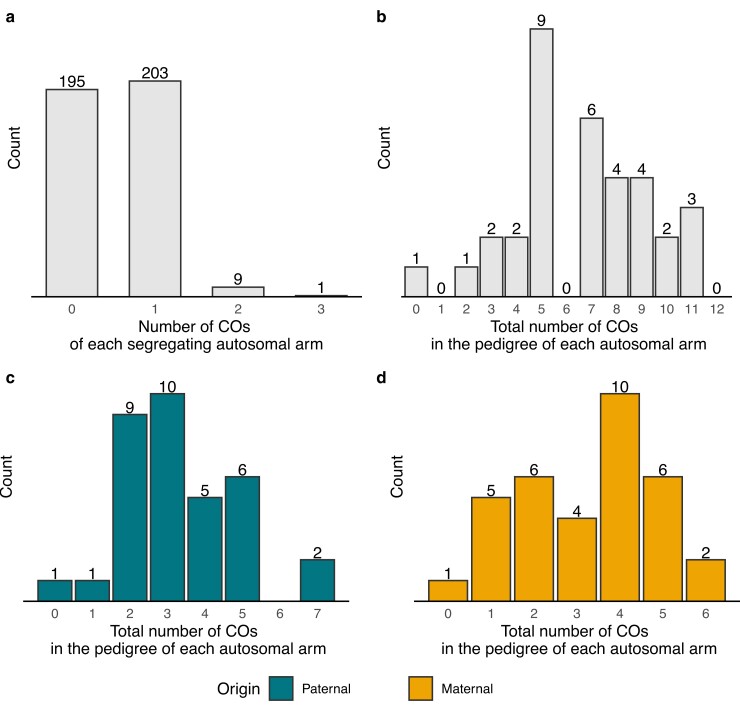
a) The distribution of COs of each segregating autosomal arm. The total number of segregating autosomal arms evaluated was 408 (34 paternal and 34 maternal autosomal arms for each of the 6 offsprings). (b–d) The distribution of the total number of COs in the pedigree of each autosomal arm (*n* = 34) considering COs of (b) both paternal and maternal origin, c) only paternal origin, and (d) only maternal origin.

We evaluated 408 autosomal arms segregating in the pedigree (34 paternal and 34 maternal chromosome arms for each of the 6 offsprings) and located 224 COs, of which 113 were paternal and 111 maternal (Supplementary Table 4-5). For the Z chromosome, we identified 1 CO in the PAR (of maternal origin) and 6 COs on the remaining part of the Z where only males recombine (Supplementary Table 4). The estimated precision of the recombination positions was generally high (mean = 557 bp; range 153–8333 bp). Of the 224 autosomal COs, 203 were single CO events on autosomal arms, 18 were double COs (i.e. 9 arms had 2 COs), and 3 formed a triple CO event (i.e. 1 arm had 3 COs; Fig. 2a). This means that 213 autosomal arms (203 + 9 + 1) had at least 1 CO whereas 195 had no CO (Fig. 2a). The number of autosomal arms with or without COs did not differ significantly (chi-square test; χ^2^ = 0.79, df = 1, *P* = 0.37). Most cases with more than 1 CO occurred on larger chromosome arms, and the shortest chromosome arm where this occurred was chromosome 15q (14.9 Mb) for which 2 COs were detected in 2 offspring (both originating from the maternal side; Supplementary Table 4). The unique case with 3 COs on a chromosome arm (in a single offspring) occurred on the long arm (q) of chromosome 4, which is one of the macrochromosomes in songbirds (Supplementary Table 4).

The total number of COs per autosomal arm segregating in the pedigree ranged between 0 and 11 (mean: 6.59) when considering both paternal and maternal COs, between 0 and 7 (mean: 3.32) considering only paternal origins, and between 0 and 6 (mean: 3.26) considering only maternal origins (Fig. 2b-d; Supplementary Table 4). This corresponds to sex-average recombination distances for the autosomal arms ranging between 0 and 91.7 cM (mean: 54.9 cM), paternal recombination distances between 0 and 116.7 cM (mean: 55.4 cM), and maternal recombination distances between 0 and 100 cM (mean: 54.4 cM). The recombination distances between paternal and maternal chromosome arms did not differ significantly (paired *t*-test; *t* = −0.21, df = 33, *P* = 0.84). For the Z chromosome, the recombination distance was 8.3 cM for the PAR (sex-average recombination) and 100 cM for the remaining part (paternal recombination).

**Fig. 3.**
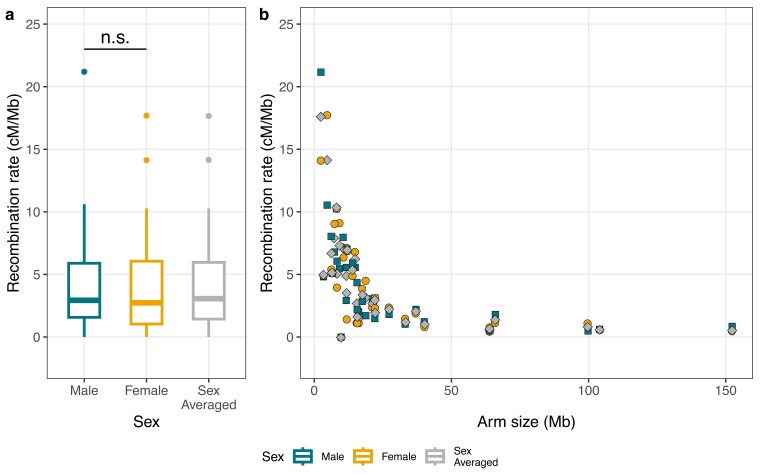
a) The distribution of male, female and sex-averaged recombination rates on autosomal arms. b) The association between the male (green rectangles), female (yellow circles) and sex-averaged (grey diamonds) recombination rates and the size of autosomal arms.

The sex-average recombination rate for the autosomal arms ranged between 0 and 17.7 cM/Mb (median: 3.06 cM/Mb), whereas the paternal rate varied between 0 and 21.2 cM/Mb (median: 2.93 cM/Mb), and the maternal rate between 0 and 17.7 cM/Mb (median: 2.73 cM/Mb) (Fig. 3a). There was no significant difference in recombination rate between the sexes (paired *t*-test; *t* = −0.16, df = 33, *P* = 0.88). There was a pronounced non-linear negative association between the recombination rate and the size of the chromosome arms (Fig. 3b). For the Z chromosome, the recombination rate was 7.58 cM/Mb for the PAR and 1.16 cM/Mb for the remaining part of the chromosome.

**Fig. 4.**
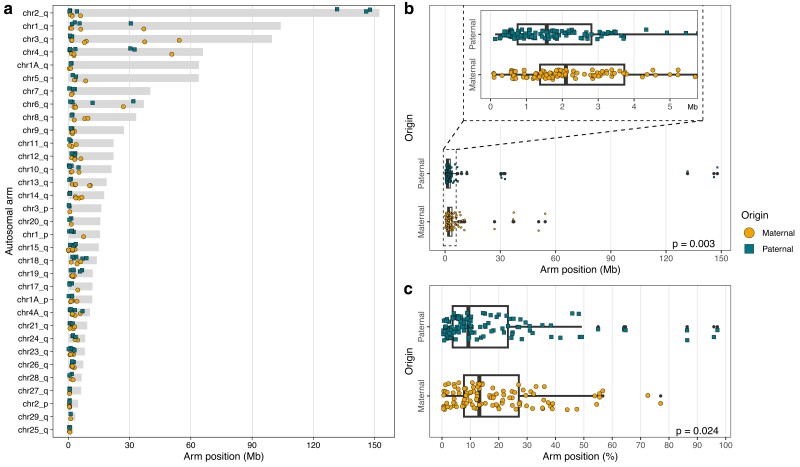
The bias of COs toward the telomeric ends of chromosomes. a) The location of CO positions of paternal and maternal origins on each chromosome arm. b) The physical distance of CO positions from the telomeric end of autosomal arms. c) The proportional distance of CO positions from the telomeric end of autosomal arms. Grey bars (a) indicate the sizes of the autosomal arms. The coloration and shape of points (a–c) indicate paternal (green squares) and maternal (yellow circles) CO events.

The position of the 224 autosomal COs showed strong bias toward the telomeric end of the autosomal arms, and this was true for both paternal and maternal chromosome arms (Fig. 4a; Supplementary Table 5). However, this bias was significantly more pronounced on paternal chromosomes, both when considering the physical distance from the telomeric end (male median position: 1.57 Mb; female median position: 2.10 Mb; Mann–Whitney U test; U = 7732, *P* = 0.003; Fig. 4b) and the proportional distance on the chromosome arms (male median position: 9.29%, female median position: 13.14%, Mann–Whitney U test; U = 7365, *P* = 0.024; Fig. 4c).

**Fig. 5.**
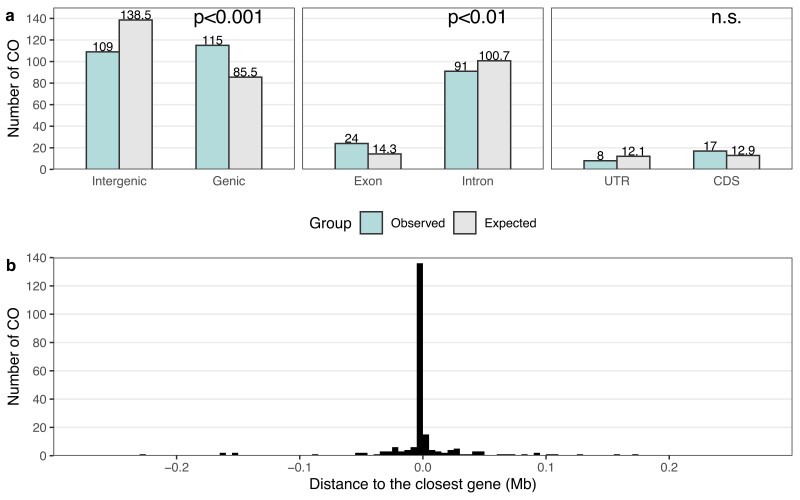
The relationship between autosomal CO events and gene features. a) Number of COs in intergenic and genic regions, in exons and introns, and in UTR and CDS (green left bars). Also given are the expected numbers based on the size of these gene features in the genome (grey right bars). b) The distribution of the distance to the closest gene for autosomal CO events.

Regarding the distribution of autosomal COs in relation to gene features, COs were underrepresented in intergenic regions and overrepresented in genic regions when accounting for the size of these regions in the genome (intergenic: 109 observed, 138.5 expected; genic: 115 observed, 85.5 expected; chi-square test; χ^2^ = 16.4, df = 1, *P* = 5.06 × 10^−5^; Fig. 5a). This was however not the case when only the chromosome ends (6-Mb sub-telomeric regions), where most COs occur (87%), were considered (chi-square test; χ^2^ = 2.71, df = 1, *P* = 0.0997; Supplementary Fig. 1). Moreover, the 115 autosomal COs in genic regions occurred more frequently in exons than in introns when accounting for the size of these regions in the genome (exons: 24 observed, 14.3 expected; introns: 91 observed, 100.7 expected; chi-square test; χ^2^ = 7.52, df = 1, *P* = 6.09 × 10^−3^; Fig. 5a). This was also the case when only the chromosome ends (6-Mb sub-telomeric regions) were considered (chi-square test; χ^2^ = 3.99, df = 1, *P* = 0.0459; Supplementary Fig. 1). Among the 24 COs in exons, 17 occurred in CDS and 8 in UTRs, which is not significantly different from an expectation based on the size of these regions (whole genome: chi-square test; χ^2^ = 2.74, df = 1, *P* = 0.0981, Fig. 5a; 6-Mb sub-telomeric regions: χ^2^ = 1.33, df = 1, *P* = 0.249, Supplementary Fig. 1).

Regarding the distance to the closest genes, 90% of the autosomal COs were located between −34 kb (upstream) and 47 kb (downstream) to the closest genes (Fig. 5b). Among the 109 intergenic COs, the distance to the closest gene was not smaller than expected by chance (permutation test; *P* = 0.932; Supplementary Fig. 2).

## Discussion

Quantifying the recombination landscape and identifying chromosome regions with varying rate of recombination can improve our understanding of the evolutionary impact of gene linkage. Additionally, it can provide insights into the mechanisms underlying the pairing and segregation of homologous chromosomes during meiosis. In this study, we analyzed whole-genome sequencing data from a 3-generation pedigree of the great reed warbler to precisely locate CO events. The high resolution of our analysis enabled us to examine the relationship between COs and various chromosomal features, as well as compare the recombination patterns between paternal and maternal chromosomes.

We observed 7 CO events on the Z chromosome, 1 in the PAR of maternal origin, and 6 in the remaining part where only males recombine. The recombination pattern on the Z chromosome was generally similar to that of similarly-sized autosomes, i.e. concentrated toward the chromosome ends. However, since the sex chromosomes segregate differently in males and females, and since recombination only occurs in the PAR in females, we will not further discuss the few cases of COs on the Z chromosome.

Our analysis focused on 408 autosomal arms segregating in the pedigree (34 arms; 12 meioses per arm among the 6 offsprings), and we identified 224 COs. The majority of autosomal arms had either 0 or 1 CO, with only 9 cases of 2 COs and 1 case of 3 COs on a single chromosome arm being detected. This implies that 52.2% of the autosomal arms had at least 1 CO. This finding aligns with 2 expectations: (1) the probability of a CO product being passed down to the offspring is 50% per chiasma, considering the presence of 2 sister chromatids with one having a CO, and (2) the hypothesis of an obligate chiasma requirement, suggesting that at least 1 chiasma per chromosome arm is necessary for proper segregation of homologous chromosomes (Coop and Przeworski 2007; Stapley *et al*. 2017). It is worth noting that the terms “crossover” and “chiasma” are sometimes used interchangeably, but that we refer to CO as the genetic recombination event and to chiasma as the cross-shaped interaction between non-sister chromatids of homologous chromosomes during meiotic prophase. The obligate chiasma requirement indicates the necessity to connect homologous chromosome arms, enabling their alignment on the spindle in metaphase I, and thereby facilitating correct segregation (Petronczki *et al*. 2003). However, there are exceptions to this requirement, most obviously among species exhibiting achiasmy, where chiasmata are absent in one of the sexes (Haldane 1922; Huxley 1928; John *et al*. 2016). Furthermore, investigations into the obligate chiasma requirement in mammals using cytogenetic and phylogenetic methods identified multiple independent shifts from 1 chiasma per chromosome arm to 1 chiasma per chromosome across the phylogenetic tree, extending the hypothesis to a minimum of 1 CO per chromosome (Dumont 2017). In this study of the great reed warbler pedigree, we detected at least 1 CO at all chromosome arms, except for chromosome arm 4p (the short arm; Supplementary Table 4) where we did not detect any CO. This could be due to chance, as zero COs can be expected to occur among 12 meiotic events (1 parental and 1 maternal meiosis for each of the 6 offsprings). Alternatively, it may indicate incomplete assembly of the telomeric end of this chromosome arm, leading to missed CO events, or that the location of the centromere is more telocentric than we estimated from the low nucleotide diversity and its location in the zebra finch. The limited occurrence of chromosome arms with multiple COs in the great reed warbler (10 cases of 213 autosomal arms with at least 1 CO) supports the notion that CO interference reduces the likelihood of additional COs (Sturtevant 1915; Muller 1916; Otto and Payseur 2019). Notably, recombination interference can be positive, resulting in fewer and/or more spaced COs (Coop and Przeworski 2007) as seems to be the case in great reed warblers, or negative, leading to a higher recombination rate than expected, which is observed in some plants and animals (Auger and Sheridan 2001; Aggarwal *et al*. 2015).

A study of recombination in humans did not only confirm the obligate chiasma requirement but also emphasized the significance of proper location of chiasmata for accurate segregation of homologous chromosomes during meiosis (Coop and Przeworski 2007). Our analysis revealed an extreme bias of COs toward the sub-telomeric regions of the chromosomes, with approximately 87% of COs occurring within approximately 6 Mb from the chromosome ends. This particular pattern has been observed in various other taxa (Vincenten *et al*. 2015; Limborg *et al*. 2016; Haenel *et al*. 2018; Sardell and Kirkpatrick 2020), including in zebra finch and collared flycatcher (Backström *et al*. 2010; Smeds *et al*. 2016), indicating a widespread phenomenon. The underlying reason for the bias of COs toward sub-telomeric regions remains an open question. One possibility is that the heterogeneous distribution of COs on chromosomes is regulated by specific DNA sequence structures or motifs. In humans, an overrepresentation of a 13-mer DNA motif has been identified as a factor inducing recombination, with the motif being recognized and bound by the zinc finger of PRDM9, a histone methyltransferase (Myers *et al*. 2008). While the *PRDM9* gene is present in many mammals, it is absent in dogs and birds, suggesting that alternative mechanisms exist (Baudat *et al*. 2010; Paigen and Petkov 2018). Other genes such as *RNF212*, *CPLX1*, and *REC8* have been repeatedly implicated in recombination events in mice, humans, cattle, and sheep (Reynolds *et al*. 2013; Kong *et al*. 2014; Ma *et al*. 2015; Johnston *et al*. 2016). A search for DNA motifs associated with recombination in estrildid finches identified a few candidate motifs, but none were found to be causative (Singhal *et al*. 2015). Likewise, we did not find evidence of overrepresented motifs around the recombination positions that we detected in the great reed warbler (H. Zhang *et al*., unpublished). Another explanation to the bias of COs toward sub-telomeric regions is that heterochromatin, which is enriched around the centromeres, prevents recombination by repressing polymerase accessibility and/or DSB formation (Ellermeier *et al*. 2010; Melamed-Bessudo and Levy 2012; Wijnker *et al*. 2013). An alternative, not mutually exclusive, explanation is that recombination initiation is telomere-guided (Higgins *et al*. 2012; Haenel *et al*. 2018; Otto and Payseur 2019). According to this hypothesis, the recombination machinery (e.g. the placement of DSBs) begins at the telomere and proceeds inward, with decreasing likelihood of additional crossovers in the central parts of chromosomes depending on the strength of recombination interference (Otto and Payseur 2019). Supporting a telomere-initiated mechanism, lower recombination rates near the middle of chromosomes, regardless of centromere location, have been observed in both animals and plants (Haenel *et al*. 2018). Another genomic feature that may be associated with recombination is the distribution of transposable elements (TEs). These might have a direct impact on recombination or indirect effects by modifying the chromatin state (Kent *et al*. 2017). In a previous study, we analyzed the distribution of repeat elements on the Z chromosome of the great reed warbler, and found higher levels of long-terminal repeats (LTRs) in the central part of the chromosome (Sigeman *et al*. 2021). However, we have not analyzed the association between autosomal COs and TEs in the present study.

Regarding the location of COs in relation to genes, we observed higher frequencies of COs in genic than in intergenic regions, and in exons than in introns. Similar results have previously been found in the collared flycatcher (Smeds *et al*. 2016) and, e.g. in the green algae *Chlamydomonas reinhardtii* (Hasan and Ness 2020). Elevated recombination rates in genic and exonic regions may be explained by such regions being enriched in euchromatin, i.e. transcriptionally active and hypomethylated DNA (Black and Whetstine 2011). In general, euchromatic regions have higher recombination rates than heterochromatic regions, possibly because histone post-transcriptional modifications that promote transcription also facilitate homogous recombination (Kim *et al*. 2005; Chen and Tyler 2022). However, the patterns of recombination and their possible mechanisms might differ between taxa as studies in human, *Drosophila* and some other insects have shown that recombination rates are generally lower within exons compared with introns and intergenic regions (Kong *et al*. 2010; Miller *et al*. 2012; Jones *et al*. 2019; i Torres *et al*. 2023).

The number of recombination events was similar between the sexes at both the chromosome and genome level in the great reed warbler, and both sexes exhibited a strong bias toward COs located near the telomeric ends of chromosomes. However, we observed that COs occurred significantly closer to the telomeres on paternal chromosome arms compared with maternal chromosome arms. Previous studies on the great reed warbler, which relied on a limited set of markers but a large multi-generation pedigree, reported approximately twice as high recombination rate in females compared with males (Hansson *et al*. 2005; Dawson *et al*. 2007). The discrepancy between these findings and our study likely arises from the fact that the markers used in the former studies were primarily located in the central regions of chromosomes, resulting in an underrepresentation of sub-telomeric CO events, which are biased toward males as observed in the present study. This emphasizes the importance of thoroughly screening the entire length of chromosomes to accurately characterize potential sex-biases in recombination. In a separate project, we have conducted genotyping using genome-wide distributed restriction site-associated DNA (RAD) markers on a multi-generational pedigree (Ponnikas *et al*. 2022). Although this dataset had a smaller number of markers (approximately 50k SNPs) compared with the present study (approximately 5M SNPs), it included multiple males and females. Preliminary analyses of the autosomal RAD data set confirmed that recombination is biased toward the end of chromosomes in both sexes, and that females have a higher frequency of recombination in the central regions of chromosomes compared with males (S. Ponnikas *et al*., unpublished). Furthermore, male-biased recombination in sub-telomeric regions has been observed in several other taxa including in birds (Backström *et al*. 2010; Smeds *et al*. 2016; Sardell and Kirkpatrick 2020), while female-biased recombination around centromeres or centrally on chromosomes has been reported in some cases (Venn *et al*. 2014; Sutherland *et al*. 2017; Sardell and Kirkpatrick 2020). Understanding the mechanisms underlying sex differences in the recombination landscape will require considering the combined effects of sex-specific centromeric and telomeric influences, and how telomere-guided initiation of recombination clusters COs in sub-telomeric regions in both sexes (Higgins *et al*. 2012; Haenel *et al*. 2018; Otto and Payseur 2019).

In conclusion, recombination plays a crucial role in evolution by generating new haplotypes that natural selection can act upon. In this study, we utilized whole-genome sequencing data of a 3-generation pedigree of the great reed warbler to locate CO positions with high precision and investigate sex-specific patterns of recombination. We found that the overall number of recombination events was similar between the sexes. However, when examining the distribution of CO positions, we discovered a pronounced bias toward the telomeric ends of the chromosomes in both sexes, with a particular strong bias on parental chromosomes. We also found that COs were more frequently occurring in genic than in intergenic regions, and in exons than in introns. Elucidating the sex-specific CO landscape in the great reed warbler provides valuable evidence to gain deeper understanding of recombination, a key mechanism in shaping the genetic diversity within populations.

## Supplementary Material

jkae150_Supplementary_Data

## Data Availability

All sequence data used for this study are accessible under BioProject ID's PRJNA970100. Supplemental material available at G3 online.

## References

[jkae150-B1] Adrion JR , Galloway JG, Kern AD. 2020. Predicting the landscape of recombination using deep learning. Mol Biol Evol. 37(6):1790–1808. doi:10.1093/molbev/msaa038. 32077950 PMC7253213

[jkae150-B2] Aggarwal DD , Rashkovetsky E, Michalak P, Cohen I, Ronin Y, Zhou D, Haddad GG, Korol AB. 2015. Experimental evolution of recombination and crossover interference in Drosophila caused by directional selection for stress-related traits. BMC Biol. 13(1):101. doi:10.1186/s12915-015-0206-5. 26614097 PMC4661966

[jkae150-B3] Arbeithuber B , Betancourt AJ, Ebner T, Tiemann-Boege I. 2015. Crossovers are associated with mutation and biased gene conversion at recombination hotspots. Proc Natl Acad Sci USA. 112(7):2109–2114. doi:10.1073/pnas.1416622112. 25646453 PMC4343121

[jkae150-B4] Auger DL , Sheridan WF. 2001. Negative crossover interference in maize translocation heterozygotes. Genetics. 159(4):1717–1726. doi:10.1093/genetics/159.4.1717. 11779809 PMC1461912

[jkae150-B5] Bachtrog D . 2013. Y-chromosome evolution: emerging insights into processes of Y-chromosome degeneration. Nat Rev Genet. 14(2):113–124. doi:10.1038/nrg3366. 23329112 PMC4120474

[jkae150-B6] Backström N , Forstmeier W, Schielzeth H, Mellenius H, Nam K, Bolund E, Webster MT, Ost T, Schneider M, Kempenaers B, et al 2010. The recombination landscape of the zebra finch *Taeniopygia guttata* genome. Genome Res. 20(4):485–495. doi:10.1101/gr.101410.109. 20357052 PMC2847751

[jkae150-B7] Baudat F , Buard J, Grey C, Fledel-Alon A, Ober C, Przeworski M, Coop G, de Massy B. 2010. PRDM9 is a Major determinant of meiotic recombination hotspots in humans and mice. Science. 327(5967):836–840. doi:10.1126/science.1183439. 20044539 PMC4295902

[jkae150-B8] Bell AD , Mello CJ, Nemesh J, Brumbaugh SA, Wysoker A, McCarroll SA. 2020. Insights into variation in meiosis from 31,228 human sperm genomes. Nature. 583(7815):259–264. doi:10.1038/s41586-020-2347-0. 32494014 PMC7351608

[jkae150-B9] Bensch S , Hasselquist D, Nielsen B, Hansson B. 1998. Higher fitness for philopatric than for immigrant males in a semi-isolated population of great reed warblers. Evolution. 52(3):877–883. doi:10.2307/2411282. 28565235

[jkae150-B10] Bergero R , Gardner J, Bader B, Yong L, Charlesworth D. 2019. Exaggerated heterochiasmy in a fish with sex-linked male coloration polymorphisms. Proc Natl Acad Sci USA. 116(14):6924–6931. doi:10.1073/pnas.1818486116. 30894479 PMC6452659

[jkae150-B11] Berset-Brändli L , Jaquiéry J, Broquet T, Ulrich Y, Perrin N. 2008. Extreme heterochiasmy and nascent sex chromosomes in European tree frogs. Proc R Soc B. 275(1642):1577–1585. doi:10.1098/rspb.2008.0298. PMC2602668 18426748

[jkae150-B12] Black JC , Whetstine JR. 2011. Chromatin landscape: methylation beyond transcription. Epigenetics. 6(1):9–15. doi:10.4161/epi.6.1.13331. 20855937 PMC3052912

[jkae150-B13] Bolger AM , Lohse M, Usadel B. 2014. Trimmomatic: a flexible trimmer for illumina sequence data. Bioinformatics. 30(15):2114–2120. doi:10.1093/bioinformatics/btu170. 24695404 PMC4103590

[jkae150-B14] Broad Institute. Picard Tools. https://broadinstitute.github.io/picard/. Accessed 21 March 2021.

[jkae150-B15] Broman KW , Murray JC, Sheffield VC, White RL, Weber JL. 1998. Comprehensive human genetic maps: individual and sex-specific variation in recombination. Am J Hum Genet. 63(3):861–869. doi:10.1086/302011. 9718341 PMC1377399

[jkae150-B16] Burt A , Bell G, Harvey PH. 1991. Sex differences in recombination. J Evolution Biol. 4(2):259–277. doi:10.1046/j.1420-9101.1991.4020259.x.

[jkae150-B17] Chan AH , Jenkins PA, Song YS. 2012. Genome-Wide fine-scale recombination rate variation in *Drosophila melanogaster*. PLoS Genet. 8(12):e1003090. doi:10.1371/journal.pgen.1003090. 23284288 PMC3527307

[jkae150-B18] Charlesworth B , Charlesworth D. 2000. The degeneration of Y chromosomes. Phil Trans R Soc Lond B. 355(1403):1563–1572. doi:10.1098/rstb.2000.0717. 11127901 PMC1692900

[jkae150-B19] Chen Z , Tyler JK. 2022. The chromatin landscape channels DNA double-strand breaks to distinct repair pathways. Front Cell Dev Biol. 10:909696. doi:10.3389/fcell.2022.909696. 35757003 PMC9213757

[jkae150-B20] Coop G , Przeworski M. 2007. An evolutionary view of human recombination. Nat Rev Genet. 8(1):23–34. doi:10.1038/nrg1947. 17146469

[jkae150-B21] Danecek P , Auton A, Abecasis G, Albers CA, Banks E, DePristo MA, Handsaker RE, Lunter G, Marth GT, Sherry ST, et al 2011. The variant call format and VCFtools. Bioinformatics. 27(15):2156–2158. doi:10.1093/bioinformatics/btr330. 21653522 PMC3137218

[jkae150-B22] Dawson DA , Akesson M, Burke T, Pemberton JM, Slate J, Hansson B. 2007. Gene order and recombination rate in homologous chromosome regions of the chicken and a passerine bird. Mol Biol Evol. 24(7):1537–1552. doi:10.1093/molbev/msm071. 17434902

[jkae150-B23] Dumont BL . 2017. Variation and evolution of the meiotic requirement for crossing over in mammals. Genetics. 205(1):155–168. doi:10.1534/genetics.116.192690. 27838628 PMC5223500

[jkae150-B24] Dumont BL , Payseur BA. 2008. Evolution of the genomic rate of recombination in mammals. Evolution. 62(2):276–294. doi:10.1111/j.1558-5646.2007.00278.x. 18067567

[jkae150-B25] Dumont BL , White MA, Steffy B, Wiltshire T, Payseur BA. 2011. Extensive recombination rate variation in the house mouse species complex inferred from genetic linkage maps. Genome Res. 21(1):114–125. doi:10.1101/gr.111252.110. 20978138 PMC3012918

[jkae150-B26] Duret L , Galtier N. 2009. Biased gene conversion and the evolution of mammalian genomic landscapes. Annu Rev Genom Hum Genet. 10(1):285–311. doi:10.1146/annurev-genom-082908-150001. 19630562

[jkae150-B27] Ellermeier C , Higuchi EC, Phadnis N, Holm L, Geelhood JL, Thon G, Smith GR. 2010. RNAi and heterochromatin repress centromeric meiotic recombination. Proc Natl Acad Sci USA. 107(19):8701–8705. doi:10.1073/pnas.0914160107. 20421495 PMC2889303

[jkae150-B28] Felsenstein J . 1974. The evolutionary advantage of recombination. Genetics. 78(2):737–756. doi:10.1093/genetics/78.2.737. 4448362 PMC1213231

[jkae150-B29] Filatov DA , Gerrard DT. 2003. High mutation rates in human and ape pseudoautosomal genes. Gene. 317:67–77. doi:10.1016/S0378-1119(03)00697-8. 14604793

[jkae150-B30] Gao F , Ming C, Hu W, Li H. 2016. New software for the fast estimation of population recombination rates (FastEPRR) in the genomic era. G3 (Bethesda). 6(6):1563–1571. doi:10.1534/g3.116.028233. 27172192 PMC4889653

[jkae150-B31] Garrison E , Kronenberg ZN, Dawson ET, Pedersen BS, Prins P. 2022. A spectrum of free software tools for processing the VCF variant call format: vcflib, bio-vcf, cyvcf2, hts-nim and slivar. PLoS Comput Biol. 18(5):e1009123. doi:10.1371/journal.pcbi.1009123. 35639788 PMC9286226

[jkae150-B32] Garrison E , Marth G. 2012. Haplotype-based variant detection from short-read sequencing. arXiv preprint 1207.3907.

[jkae150-B33] Gergelits V , Parvanov E, Simecek P, Forejt J. 2021. Chromosome-wide characterization of meiotic noncrossovers (gene conversions) in mouse hybrids. Genetics. 217(1):iyaa013. doi:10.1093/genetics/iyaa013. 33683354 PMC8045703

[jkae150-B34] Groenen MAM , Wahlberg P, Foglio M, Cheng HH, Megens H-J, Crooijmans RPMA, Besnier F, Lathrop M, Muir WM, Wong GK-S, et al 2009. A high-density SNP-based linkage map of the chicken genome reveals sequence features correlated with recombination rate. Genome Res. 19(3):510–519. doi:10.1101/gr.086538.108. 19088305 PMC2661806

[jkae150-B35] Haenel Q , Laurentino TG, Roesti M, Berner D. 2018. Meta-analysis of chromosome-scale crossover rate variation in eukaryotes and its significance to evolutionary genomics. Mol Ecol. 27(11):2477–2497. doi:10.1111/mec.14699. 29676042

[jkae150-B36] Haldane J . 1920. Note on a case of linkage in *Paratettix*. J Genet. 10(1):47–51. doi:10.1007/BF02983522.

[jkae150-B37] Haldane JB . 1922. Sex ratio and unisexual sterility in hybrid animals. J Genet. 12(2):101–109. doi:10.1007/BF02983075.

[jkae150-B38] Halldorsson BV , Palsson G, Stefansson OA, Jonsson H, Hardarson MT, Eggertsson HP, Gunnarsson B, Oddsson A, Halldorsson GH, Zink F, et al 2019. Characterizing mutagenic effects of recombination through a sequence-level genetic map. Science. 363(6425):eaau1043. doi:10.1126/science.aau1043. 30679340

[jkae150-B39] Hansson B , Åkesson M, Slate J, Pemberton JM. 2005. Linkage mapping reveals sex-dimorphic map distances in a passerine bird. Proc R Soc B. 272(1578):2289–2298. doi:10.1098/rspb.2005.3228. PMC1560182 16191642

[jkae150-B40] Hansson B , Sigeman H, Stervander M, Tarka M, Ponnikas S, Strandh M, Westerdahl H, Hasselquist D. 2018. Contrasting results from GWAS and QTL mapping on wing length in great reed warblers. Mol Ecol Resour. 18(4):867–876. doi:10.1111/1755-0998.12785. 29658173

[jkae150-B41] Hasan AR , Ness RW. 2020. Recombination rate variation and infrequent sex influence genetic diversity in *Chlamydomonas reinhardtii*. Genome Biol Evol. 12(4):370–380. doi:10.1093/gbe/evaa057. 32181819 PMC7186780

[jkae150-B42] Hasselquist D . 1998. Polygyny in great reed warblers: a long-term study of factors contributing to male fitness. Ecology. 79(7):2376–2390. doi:10.1890/0012-9658(1998)079[2376:PIGRWA]2.0.CO;2.

[jkae150-B43] Helbig AJ , Seibold I. 1999. Molecular phylogeny of palearctic–African *Acrocephalus* and *Hippolais* warblers (aves: sylviidae). Mol Phylogenet Evol. 11(2):246–260. doi:10.1006/mpev.1998.0571. 10191069

[jkae150-B44] Hellmann I , Ebersberger I, Ptak SE, Pääbo S, Przeworski M. 2003. A neutral explanation for the correlation of diversity with recombination rates in humans. Am J Hum Genet. 72(6):1527–1535. doi:10.1086/375657. 12740762 PMC1180312

[jkae150-B45] Higgins JD , Perry RM, Barakate A, Ramsay L, Waugh R, Halpin C, Armstrong SJ, Franklin FCH. 2012. Spatiotemporal asymmetry of the meiotic program underlies the predominantly distal distribution of meiotic crossovers in barley. Plant Cell. 24(10):4096–4109. doi:10.1105/tpc.112.102483. 23104831 PMC3517238

[jkae150-B46] Huang S-W , Friedman R, Yu N, Yu A, Li W-H. 2005. How strong is the mutagenicity of recombination in mammals? Mol Biol Evol. 22(3):426–431. doi:10.1093/molbev/msi025. 15496551

[jkae150-B47] Huxley J . 1928. Sexual difference of linkage in *Gammarus chevreuxi*. J Genet. 20(2):145–156. doi:10.1007/BF02983136.

[jkae150-B48] I Torres A , Höök L, Näsvall K, Shipilina D, Wiklund C, Vila R, Pruisscher P, Backström N. 2023. The fine-scale recombination rate variation and associations with genomic features in a butterfly. Genome Res. 33:810–823. doi:10.1101/gr.277414.122. 37308293 PMC10317125

[jkae150-B49] John A , Vinayan K, Varghese J. 2016. Achiasmy: male fruit flies are not ready to mix. Front Cell Dev Biol. 4:75. doi:10.3389/fcell.2016.00075. 27486580 PMC4949207

[jkae150-B50] Johnston SE , Bérénos C, Slate J, Pemberton JM. 2016. Conserved genetic architecture underlying individual recombination rate variation in a wild population of soay sheep (*Ovis aries*). Genetics. 203(1):583–598. doi:10.1534/genetics.115.185553. 27029733 PMC4858801

[jkae150-B51] Johnston SE , Huisman J, Ellis PA, Pemberton JM. 2017. A high-density linkage map reveals sexual dimorphism in recombination landscapes in red deer (*Cervus elaphus*). G3 (Bethesda). 7:2859–2870. doi:10.1534/g3.117.044198. 28667018 PMC5555489

[jkae150-B52] Jones JC , Wallberg A, Christmas MJ, Kapheim KM, Webster MT. 2019. Extreme differences in recombination rate between the genomes of a solitary and a social bee. Mol Biol Evol. 36:2277–2291. doi:10.1093/molbev/msz130. 31143942

[jkae150-B53] Kassambara A . 2022. rstatix: Pipe-friendly framework for basic statistical tests. R package version 0.7.1.

[jkae150-B54] Kawakami T , Smeds L, Backström N, Husby A, Qvarnström A, Mugal CF, Olason P, Ellegren H. 2014. A high-density linkage map enables a second-generation collared flycatcher genome assembly and reveals the patterns of avian recombination rate variation and chromosomal evolution. Mol Ecol. 23:4035–4058. doi:10.1111/mec.12810. 24863701 PMC4149781

[jkae150-B55] Kent TV , Uzunović J, Wright SI. 2017. Coevolution between transposable elements and recombination. Phil. Trans. R. Soc. B. 372:20160458. doi:10.1098/rstb.2016.0458. 29109221 PMC5698620

[jkae150-B56] Kim J-S , Islam-Faridi MN, Klein PE, Stelly DM, Price HJ, Klein RR, Mullet JE. 2005. Comprehensive molecular cytogenetic analysis of sorghum genome architecture: distribution of euchromatin, heterochromatin, genes and recombination in comparison to rice. Genetics. 171:1963–1976. doi:10.1534/genetics.105.048215. 16143604 PMC1456119

[jkae150-B57] Knief U , Forstmeier W. 2016. Mapping centromeres of microchromosomes in the zebra finch (*Taeniopygia guttata*) using half-tetrad analysis. Chromosoma. 125:757–768. doi:10.1007/s00412-015-0560-7. 26667931 PMC5023761

[jkae150-B58] Kohl KP , Singh ND. 2018. Experimental evolution across different thermal regimes yields genetic divergence in recombination fraction but no divergence in temperature associated plastic recombination. Evolution. 72:989–999. doi:10.1111/evo.13454. 29468654

[jkae150-B59] Koleček J , Procházka P, El-Arabany N, Tarka M, Ilieva M, Hahn S, Honza M, de la Puente J, Bermejo A, Gürsoy A, et al 2016. Cross-continental migratory connectivity and spatiotemporal migratory patterns in the great reed warbler. J Avian Biol. 47:756–767. doi:10.1111/jav.00929.

[jkae150-B60] Kong A , Barnard J, Gudbjartsson DF, Thorleifsson G, Jonsdottir G, Sigurdardottir S, Richardsson B, Jonsdottir J, Thorgeirsson T, Frigge ML, et al 2004. Recombination rate and reproductive success in humans. Nat Genet. 36:1203–1206. doi:10.1038/ng1445. 15467721

[jkae150-B61] Kong A , Thorleifsson G, Frigge ML, Masson G, Gudbjartsson DF, Villemoes R, Magnusdottir E, Olafsdottir SB, Thorsteinsdottir U, Stefansson K. 2014. Common and low-frequency variants associated with genome-wide recombination rate. Nat Genet. 46:11–16. doi:10.1038/ng.2833. 24270358

[jkae150-B62] Kong A , Thorleifsson G, Gudbjartsson DF, Masson G, Sigurdsson A, Jonasdottir A, Walters GB, Jonasdottir A, Gylfason A, Kristinsson KT, et al 2010. Fine-scale recombination rate differences between sexes, populations and individuals. Nature. 467:1099–1103. doi:10.1038/nature09525. 20981099

[jkae150-B63] Lemke HW , Tarka M, Klaassen RHG, Åkesson M, Bensch S, Hasselquist D, Hansson B. 2013. Annual cycle and migration strategies of a trans-saharan migratory songbird: a geolocator study in the great reed warbler. PLoS One. 8:e79209. doi:10.1371/journal.pone.0079209. 24205374 PMC3799637

[jkae150-B64] Li H . 2013. Aligning sequence reads, clone sequences and assembly contigs with BWA-MEM. arXiv preprint 1303.3997.

[jkae150-B65] Limborg MT , McKinney GJ, Seeb LW, Seeb JE. 2016. Recombination patterns reveal information about centromere location on linkage maps. Mol Ecol Resour. 16:655–661. doi:10.1111/1755-0998.12484. 26561199

[jkae150-B66] Ma L , O'Connell JR, VanRaden PM, Shen B, Padhi A, Sun C, Bickhart DM, Cole JB, Null DJ, Liu GE, et al 2015. Cattle sex-specific recombination and genetic control from a large pedigree analysis. PLoS Genet. 11:e1005387. doi:10.1371/journal.pgen.1005387. 26540184 PMC4634960

[jkae150-B67] Maddox JF , Davies KP, Crawford AM, Hulme DJ, Vaiman D, Cribiu EP, Freking BA, Beh KJ, Cockett NE, Kang N, et al 2001. An enhanced linkage map of the sheep genome comprising more than 1000 loci. Genome Res. 11:1275–1289. doi:10.1101/gr.135001. 11435411 PMC311104

[jkae150-B68] Malinovskaya LP , Tishakova K, Shnaider EP, Borodin PM, Torgasheva AA. 2020. Heterochiasmy and sexual dimorphism: the case of the barn swallow (Hirundo rustica, hirundinidae, aves). Genes (Basel). 11:1119. doi:10.3390/genes11101119. 32987748 PMC7650650

[jkae150-B69] McVean G , Auton A. 2007. LDhat 2.1: a Package for the Population Genetic Analysis of Recombination. Oxford, OX1 3TG, UK: Department of Statistics.

[jkae150-B70] Melamed-Bessudo C , Levy AA. 2012. Deficiency in DNA methylation increases meiotic crossover rates in euchromatic but not in heterochromatic regions in *Arabidopsis*. Proc Natl Acad Sci USA. 109:E981–E988. doi:10.1073/pnas.1120742109. 22460791 PMC3341010

[jkae150-B71] Miller DE , Takeo S, Nandanan K, Paulson A, Gogol MM, Noll AC, Perera AG, Walton KN, Gilliland WD, Li H, et al 2012. A whole-chromosome analysis of meiotic recombination in Drosophila melanogaster. G3 (Bethesda). 2:249–260. doi:10.1534/g3.111.001396. 22384403 PMC3284332

[jkae150-B72] Morgan TH . 1910. Sex limited inheritance in *Drosophila*. Science. 32:120–122. doi:10.1126/science.32.812.120. 17759620

[jkae150-B73] Morton N , Rao D, Yee S. 1976. An inferred chiasma map of *Drosophila melanogaster*. Heredity (Edinb). 37:405–411. doi:10.1038/hdy.1976.105. 827535

[jkae150-B74] Mugal CF , Weber CC, Ellegren H. 2015. GC-biased gene conversion links the recombination landscape and demography to genomic base composition: GC-biased gene conversion drives genomic base composition across a wide range of species. BioEssays. 37:1317–1326. doi:10.1002/bies.201500058. 26445215

[jkae150-B75] Muller HJ . 1916. The mechanism of crossing-over. Am Nat. 50:193–221. doi:10.1086/279534.

[jkae150-B76] Myers S , Freeman C, Auton A, Donnelly P, McVean G. 2008. A common sequence motif associated with recombination hot spots and genome instability in humans. Nat Genet. 40:1124–1129. doi:10.1038/ng.213. 19165926

[jkae150-B77] Otto SP . 2021. Selective interference and the evolution of sex. J Hered. 112:9–18. doi:10.1093/jhered/esaa026. 33047117

[jkae150-B78] Otto SP , Payseur BA. 2019. Crossover interference: shedding light on the evolution of recombination. Annu Rev Genet. 53:19–44. doi:10.1146/annurev-genet-040119-093957. 31430178 PMC8715713

[jkae150-B79] Paigen K , Petkov PM. 2018. PRDM9 and its role in genetic recombination. Trends Genet. 34:291–300. doi:10.1016/j.tig.2017.12.017. 29366606 PMC5878713

[jkae150-B80] Peñalba JV , Wolf JBW. 2020. From molecules to populations: appreciating and estimating recombination rate variation. Nat Rev Genet. 21:476–492. doi:10.1038/s41576-020-0240-1. 32472059

[jkae150-B81] Petronczki M , Siomos MF, Nasmyth K. 2003. Un menage a quatre: the molecular biology of chromosome segregation in meiosis. Cell. 112:423–440. doi:10.1016/S0092-8674(03)00083-7. 12600308

[jkae150-B82] Ponnikas S , Sigeman H, Lundberg M, Hansson B. 2022. Extreme variation in recombination rate and genetic diversity along the Sylvioidea neo-sex chromosome. Mol Ecol. 31:3566–3583. doi:10.1111/mec.16532. 35578784 PMC9327509

[jkae150-B83] Provost K , Shue SY, Forcellati M, Smith BT. 2022. The genomic landscapes of desert birds form over multiple time scales. Mol Biol Evol. 39:msac200. doi:10.1093/molbev/msac200. 36134537 PMC9577548

[jkae150-B84] Quinlan AR , Hall IM. 2010. BEDTools: a flexible suite of utilities for comparing genomic features. Bioinformatics. 26:841–842. doi:10.1093/bioinformatics/btq033. 20110278 PMC2832824

[jkae150-B85] R Core Team . 2022. R: A Language and Environment for Statistical Computing. Vienna, Austria: R Foundation for Statistical Computing.

[jkae150-B86] Reynolds A , Qiao H, Yang Y, Chen JK, Jackson N, Biswas K, Holloway JK, Baudat F, de Massy B, Wang J, et al 2013. RNF212 is a dosage-sensitive regulator of crossing-over during mammalian meiosis. Nat Genet. 45:269–278. doi:10.1038/ng.2541. 23396135 PMC4245152

[jkae150-B87] Ritz KR , Noor MA, Singh ND. 2017. Variation in recombination rate: adaptive or not? Trends Genet. 33:364–374. doi:10.1016/j.tig.2017.03.003. 28359582

[jkae150-B88] Robinson WP . 1996. The extent, mechanism, and consequences of genetic variation, for recombination rate. Am J Hum Genet. 59:1175–1183. 8940261 PMC1914866

[jkae150-B89] Sardell JM , Kirkpatrick M. 2020. Sex differences in the recombination landscape. Am Nat. 195:361–379. doi:10.1086/704943. 32017625 PMC7537610

[jkae150-B90] Sigeman H , Strandh M, Proux-Wéra E, Kutschera VE, Ponnikas S, Zhang H, Lundberg M, Soler L, Bunikis I, Tarka M, et al 2021. Avian neo-sex chromosomes reveal dynamics of recombination suppression and W degeneration. Mol Biol Evol. 38:5275–5291. doi:10.1093/molbev/msab277. 34542640 PMC8662655

[jkae150-B91] Singhal S , Leffler EM, Sannareddy K, Turner I, Venn O, Hooper DM, Strand AI, Li Q, Raney B, Balakrishnan CN, et al 2015. Stable recombination hotspots in birds. Science. 350:928–932. doi:10.1126/science.aad0843. 26586757 PMC4864528

[jkae150-B92] Sjöberg S , Malmiga G, Nord A, Andersson A, Bäckman J, Tarka M, Willemoes M, Thorup K, Hansson B, Alerstam T, et al 2021. Extreme altitudes during diurnal flights in a nocturnal songbird migrant. Science. 372:646–648. doi:10.1126/science.abe7291. 33958477

[jkae150-B93] Smeds L , Mugal CF, Qvarnström A, Ellegren H. 2016. High-Resolution mapping of crossover and non-crossover recombination events by whole-genome Re-sequencing of an avian pedigree. PLoS Genet. 12:e1006044. doi:10.1371/journal.pgen.1006044. 27219623 PMC4878770

[jkae150-B94] Stapley J , Feulner PGD, Johnston SE, Santure AW, Smadja CM. 2017. Variation in recombination frequency and distribution across eukaryotes: patterns and processes. Phil Trans R Soc B. 372:20160455. doi:10.1098/rstb.2016.0455. 29109219 PMC5698618

[jkae150-B95] Sætre CLC , Eroukhmanoff F, Rönkä K, Kluen E, Thorogood R, Torrance J, Tracey A, Chow W, Pelan S, Howe K, et al 2021. A chromosome-level genome assembly of the reed warbler (*Acrocephalus scirpaceus*). Genome Biol Evol. 13:evab212. doi:10.1093/gbe/evab212. 34499122 PMC8459166

[jkae150-B96] Stump AD , Fitzpatrick MC, Lobo NF, Traoré S, Sagnon N, Costantini C, Collins FH, Besansky NJ. 2005. Centromere-proximal differentiation and speciation in *Anopheles gambiae*. Proc Natl Acad Sci USA. 102:15930–15935. doi:10.1073/pnas.0508161102. 16247019 PMC1276105

[jkae150-B97] Sturtevant AH . 1915. The behavior of the chromosomes as studied through linkage. Z Indukt Abstammungs- Vererbungsl. 13:234–287.

[jkae150-B98] Sutherland BJ , Rico C, Audet C, Bernatchez L. 2017. Sex chromosome evolution, heterochiasmy, and physiological QTL in the salmonid brook charr Salvelinus fontinalis. G3 (Bethesda). 7:2749–2762. doi:10.1534/g3.117.040915. 28626004 PMC5555479

[jkae150-B99] Tan A , Abecasis GR, Kang HM. 2015. Unified representation of genetic variants. Bioinformatics. 31:2202–2204. doi:10.1093/bioinformatics/btv112. 25701572 PMC4481842

[jkae150-B100] Tarka M , Åkesson M, Hasselquist D, Hansson B. 2014. Intralocus sexual conflict over wing length in a wild migratory bird. Am Nat. 183:62–73. doi:10.1086/674072. 24334736

[jkae150-B101] Venn O , Turner I, Mathieson I, de Groot N, Bontrop R, McVean G. 2014. Strong male bias drives germline mutation in chimpanzees. Science. 344:1272–1275. doi:10.1126/science.344.6189.1272. 24926018 PMC4746749

[jkae150-B102] Vincenten N , Kuhl L-M, Lam I, Oke A, Kerr AR, Hochwagen A, Fung J, Keeney S, Vader G, Marston AL. 2015. The kinetochore prevents centromere-proximal crossover recombination during meiosis. eLife. 4:e10850. doi:10.7554/eLife.10850. 26653857 PMC4749563

[jkae150-B103] Wellenreuther M , Sánchez-Guillén RA, Cordero-Rivera A, Svensson EI, Hansson B. 2013. Male-biased recombination in odonates: insights from a linkage map of the damselfly *Ischnura elegans*. J Genet. 92:115–119. doi:10.1007/s12041-013-0219-1. 23640414

[jkae150-B104] Westerdahl H , Mellinger S, Sigeman H, Kutschera VE, Proux-Wéra E, Lundberg M, Weissensteiner M, Churcher A, Bunikis I, Hansson B, et al 2022. The genomic architecture of the passerine MHC region: high repeat content and contrasting evolutionary histories of single copy and tandemly duplicated MHC genes. Mol Ecol Resour. 22:2379–2395. doi:10.1111/1755-0998.13614. 35348299

[jkae150-B105] Wickham H . 2011. ggplot2. Wiley interdisciplinary reviews: computational statistics. 3(2):180–185.

[jkae150-B106] Wickham H , Averick M, Bryan J, Chang W, McGowan L, François R, Grolemund G, Hayes A, Henry L, Hester J, et al 2019. Welcome to the tidyverse. J Open Source Softw. 4:1686. doi:10.21105/joss.01686.

[jkae150-B107] Wijnker E , Velikkakam James G, Ding J, Becker F, Klasen JR, Rawat V, Rowan BA, de Jong DF, de Snoo CB, Zapata L, et al 2013. The genomic landscape of meiotic crossovers and gene conversions in *Arabidopsis thaliana*. eLife. 2:e01426. doi:10.7554/eLife.01426. 24347547 PMC3865688

[jkae150-B108] Zhang H , Hansson B. 2023. RecView: an interactive R application for locating recombination positions using pedigree data. BMC Genomics. 24:1–10. doi:10.1186/s12864-023-09807-2. 38007417 PMC10676570

