# Supplementary material for: Male-biased recombination at chromosome ends in a songbird revealed by precisely mapping crossover positions
Source: G3 (Bethesda). 2024 Jul 10;14(9):jkae150. doi: 10.1093/g3journal/jkae150 (PMC11373659; doi:10.1093/g3journal/jkae150)
Supplement: jkae150_Supplementary_Data [file jkae150_supplementary_data.zip › Supplementary_Figures_G3-2024-404836.docx]

**
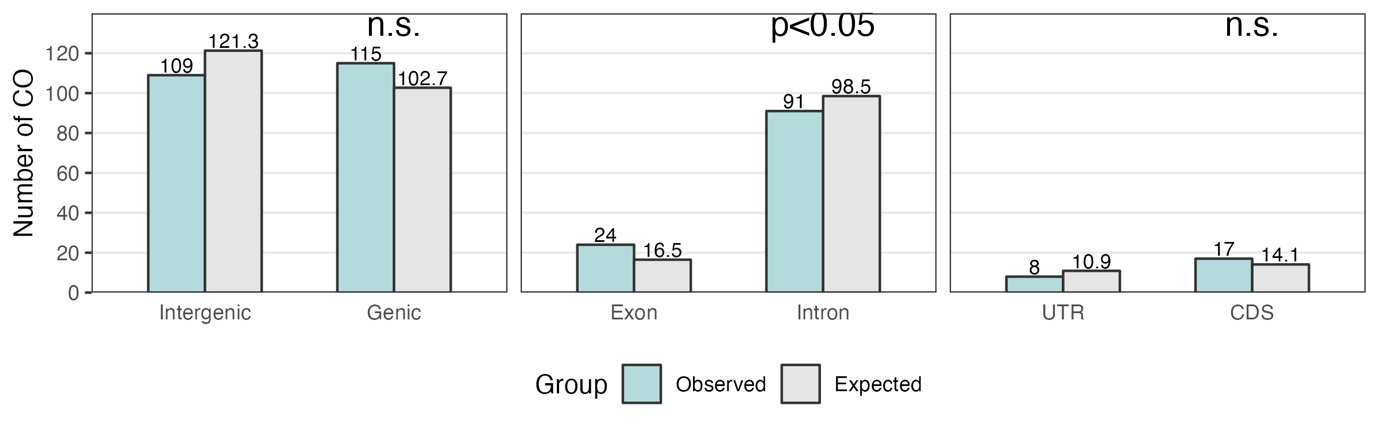
**

**Figure S1.** The relationship between autosomal CO events and gene features. Number of COs in intergenic and genic regions, in exons and introns, and in UTR and CDS (green bars). Also given are the expected numbers based on the size of these gene features within 6 Mb of the end of chromosome arms (grey bars).

**
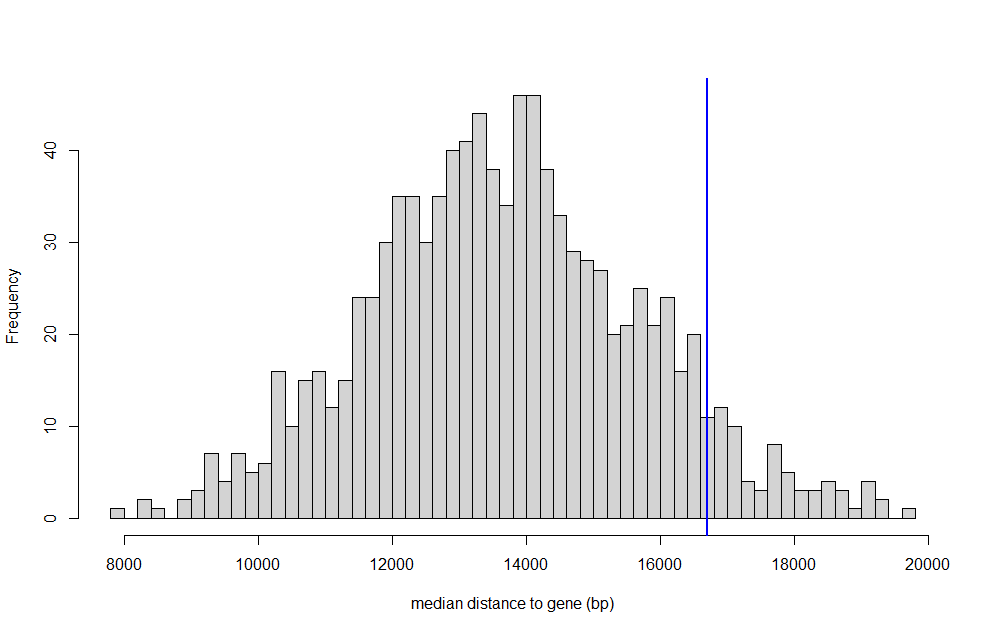
**

**Figure S2**. Null distribution of median gene distances obtained from 1,000 iterations of randomly selecting positions in intergenic intervals. The blue line represents the observed value (16,705 bp) among the 109 intergenic COs, which is higher than 93.2 % of the permuted values.
